# The Neurobiology of Social Adversity and Disparity: Analyzing Social-Stress and Health Inequities

**DOI:** 10.1093/geroni/igaf122.3424

**Published:** 2025-12-31

**Authors:** Christina Anurum-Anyanwu, Nasim Ferdows

**Affiliations:** Institute for Health Equity and Social Justice, Union, New Jersey, United States; Northeastern University, Boston, Massachusetts, United States

## Abstract

Social stress, stemming from institutional and interpersonal hardships, has been shown to diminish long-term health and increase mortality. These adverse health outcomes are strongly linked to dysregulation in neurochemical stress pathways and can include anxiety, depression, cardiovascular disease, metabolic disorders, and immune deficiency. As exposure to such adversity is often manifested in social inequities, the focus of this analysis, aims to examine how this harm is further exacerbated within historically marginalized populations especially amongst racial and ethnic minorities. This study explores the neurobiological and psychosocial mechanisms underlying the effects of social and environmental inequities on long-term health in disenfranchised communities.

